# FloSeal Related Small Bowel Obstruction: A Word of Caution

**DOI:** 10.1155/cris/1127650

**Published:** 2026-11-04

**Authors:** Gavin J. Carmichael, Jessica E. Hanna, Daniel Ng Ying Kin, Mathew O. Jacob

**Affiliations:** ^1^ Adelaide University Adelaide South Australia Australia adelaide.edu.au; ^2^ Grampians Health Ballarat Victoria Australia; ^3^ Grampians Research Initiative Ballarat Victoria Australia; ^4^ Deakin University Ballarat Victoria Australia deakin.edu.au; ^5^ University of Melbourne Melbourne Victoria Australia unimelb.edu.au

## Abstract

Topical haemostatic agents such as FloSeal are widely used to manage low‐grade bleeding in areas where conventional haemostatic techniques may risk damage to critical structures or are unsuccessful. While effective, these agents can cause unexpected complications. This case report highlights small bowel obstruction (SBO) as a rare but important adverse outcome following FloSeal use in colorectal surgery. A 55‐year‐old male underwent laparoscopic right hemicolectomy for hepatic flexure adenocarcinoma. Due to raw surface oozing near Gerota’s fascia and proximity to the duodenum, FloSeal was applied intra‐operatively. On postoperative day 3, the patient developed nausea, absent flatus and bowel sounds and a rising C‐reactive protein (CRP) (366 mg/L), progressing to 540 mg/L by day 4. CT imaging showed ileus with an intact anastomosis and no closed‐loop features. Exploratory laparotomy identified a transition point where the distal ileum was adhered to residual FloSeal, resulting in a high‐grade obstruction. Following adhesiolysis and irrigation, the patient recovered uneventfully. Normal lactate and a negative septic screen argued against bacterial translocation with systemic sepsis as the driver of the CRP rise, suggesting instead a localised inflammatory response to mechanical obstruction. We frame this as a preventable, surgical technique‐related complication rather than an intrinsic hazard of FloSeal, arising from a deliberate deviation from manufacturer instructions to remove excess material. Surgeons should maintain a high index of suspicion for SBO in patients with rising CRP and no radiological leak, and should clearly document whenever haemostatic material is deliberately retained.

## 1. Introduction

The gold standard for controlling mild haemorrhage and oozing is pressure, bipolar diathermy or suture ligation [1]. However, biotechnological advancements have introduced haemostatic agents as adjuncts to traditional methods. These products, typically comprised of fibrin, thrombin and gelatin, are designed for application to areas of mild bleeding [2, 3]. Especially around critical structures that otherwise could be damaged using traditional methods of haemostasis. Despite their benefits, these agents can have adverse effects, including the rare outcome of small bowel obstruction (SBO).

This report describes a rare case of SBO in the post‐operative period caused by FloSeal use in colorectal surgery, with CT imaging to illustrate the findings. The case highlights the importance of appropriate application techniques and adherence to manufacturer guidelines to minimise the risk of complications associated with haemostatic agents.

## 2. Case

A 55‐year‐old man underwent a laparoscopic right hemicolectomy for a biopsy confirmed adenocarcinoma of the hepatic flexure, with a background of sarcoidosis. The operation was challenging due to a high‐riding, subhepatic proximal transverse colon and hepatic flexure, complicated by extravasation of the tattoo from the bowel. The resultant dissection was off‐plane, leading to a raw surface ooze on the upper edge of Gerotas’ fascia. A limited wash of the raw surface was performed to clear up any blood. The FloSeal surface was not irrigated. Due to its proximity to the duodenum, 5 mL of FloSeal was applied to this region with a good haemostatic effect before closure. A limited wash was then performed to clear residual blood from the surrounding field, but the FloSeal site itself was deliberately excluded from this irrigation: to ensure adequate vessel tamponade, a small amount was left at the oozing site. Conventional measures had already proven insufficient, and the operating surgeon judged that formally wiping away or irrigating the excess risked disturbing the clot and re‐starting bleeding from the friable, off‐plane surface.

On post‐operative day 3, the patient developed persistent nausea and diffuse abdominal pain without focal peritonism, with no flatus passed and no bowel sounds audible. He remained afebrile and had a normal blood pressure, with transient sinus tachycardia that improved with intravenous fluids. C‐reactive protein (CRP) was markedly elevated at 366 mg/L, with a lactate of 1.4 mmol/L, prompting a CT scan that showed ileus with intact anastomosis (Figure 1). This scan was reported as: small bowel loops were dilated with a paucity of distal colonic gas, in keeping with ileus; no discrete transition point, calibre change, or closed‐loop configuration, and the residual FloSeal itself was not identified as a discrete radiological abnormality. It was retrospectively identified on CT post‐laparotomy, demonstrating hindsight bias and reflects the diagnostic dilemma that the clinicians faced. This was consistent with, though did not exclude, evolving obstruction, and likely contributed to the initial impression of ileus rather than mechanical SBO. By post‐operative day 4, the CRP had risen to 540 mg/L, prompting an immediate surgical exploration for a suspected anastomotic leak.

**Figure 1:**
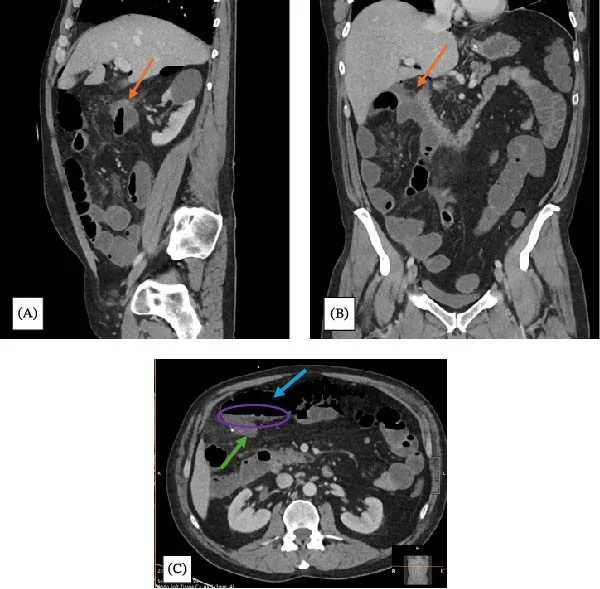
(A) Sagittal CT abdomen and pelvis. (B) Coronal CT abdomen and pelvis. (C) Axial CT abdomen and pelvis. Orange arrows: FloSeal remnant. Blue arrow: large bowel conduit. Green arrow: small bowel conduit. Purple circle: intact ileocolic anastomosis.

An upper midline incision was performed. On entry, a haemoserous fluid was encountered without enteric content or purulent material. The anastomosis appeared intact, but a high‐grade small bowel dilatation was noted with a transition point at the distal ileum, ~20 cm from the anastomosis. The involved loop was acutely kinked and adhered to the site of FloSeal application. The remnant FloSeal was ~3 mL in volume, plastering the ileum to the raw surface of dissection and appearing almost crystallised. The adhesion was carefully finger‐dissected, and the remnant FloSeal was thoroughly irrigated and removed. No further surgical intervention was required, and bowel function returned rapidly, day 2 post take back. Follow‐up was performed at 2‐weeks, 6‐weeks and 1 year post‐admission with no further recurrence of SBO. Written consent was gained for the use and publication of this case.

## 3. Discussion

SBO is an uncommon but important complication following the use of haemostatic agents in surgery. While these agents are highly effective in controlling bleeding, their interaction with peritoneal surfaces and adjacent structures poses a risk for adhesion formation and subsequent obstruction. Several case reports [4–8] have described similar complications associated with gelatin‐based products, such as FloSeal, as well as oxidised regenerated cellulose (Surgicel), see Supporting Information Table 1 for a comparison table. These agents, when left unabsorbed or inadequately irrigated, may contribute to adhesion development, mechanical obstruction, or inflammatory reactions, leading to fibrosis [9]. Agents containing gelatin, such as FloSeal, have been associated with adhesion formation [6–8], and delayed local tissue healing [10] a hypothesis for why is that they persist in tissues for 6–8 weeks [6, 11, 12], potentially prolonging inflammation.

CRP is an acute‐phase reactant that rises in response to infection, inflammation, or tissue injury and has multiple differentials that could cause an elevation (see Table 1 for causes). In this patient, the intra‐operative finding of a discrete, easily dissectible adhesive band directly incorporating the retained FloSeal, situated away from both the anastomosis and any port site, combined with the absence of any other identifiable cause at laparotomy, made the haemostatic agent the most plausible single contributor. Causality remains presumptive however the clinical reasoning listed in Table 1 aided in excluding other causes.

**Table 1:** Causes of CRP elevation and clinical reason for exclusion.

Causes	Clinical reason for exclusion
Post‐operative adhesions	The adhesion found at laparotomy was a single, easily finger‐dissectible band that directly incorporated the retained material and was situated away from any port site
Bowel handling injury	The transition point was 20 cm from the anastomosis and from the area of most extensive mobilisation, at the specific location where FloSeal had been applied
Mesenteric or internal hernial defect	There was no defect identified, and the small bowel loop was tethered to the retained material
Anastomotic leak/narrowed anastomosis	The anastomosis was visually intact, and the transition point was proximal to its location
Intra‐abdominal collection/abscess	The absence of enteric content, pus, or a defined collection on entry and exploration

CRP kinetics post‐operatively provide important details of recovery and complication risk. Published literature has demonstrated that CRP typically peaks at 48–72 h post‐operatively and then declines [10]. Predictive studies of CRP and anastomotic leak have shown day 3 cut‐offs of ~175–185 mg/L for predicting complications, including anastomotic leak [13]. Our patient’s CRP of 366 mg/L on day 3 was already more than double the published day‐3 threshold associated with major complications, and rather than beginning the expected decline it continued to rise to 540 mg/L by day 4. This was the opposite of the trajectory seen in an uncomplicated recovery process.

One proposed mechanism linking CRP elevation to SBO is bacterial translocation: studies suggest that CRP rises correlate with prolonged stasis, which disrupts mucosal integrity and allows bacteria to pass into the mesenteric lymph nodes, liver, spleen and bloodstream [14–16]. This process triggers a systemic inflammatory response, contributing to the rising CRP levels. Experimental studies [15, 17] have demonstrated that CRP levels increase with the severity of bacterial translocation in acute intestinal obstruction. In this patient, however, serum lactate was 1.4 mmol/L (normal) and a septic screen, including blood cultures, was negative, arguing against established bacterial translocation with overt systemic sepsis as the dominant driver. The white cell count fell substantially over the first three postoperative days following adhesiolysis, paralleling rapid clinical improvement. Taken together, these findings suggest that the marked CRP elevation more likely reflected a localised inflammatory response to mechanical obstruction and bowel wall congestion, rather than established bacterial translocation with systemic sepsis.

The risk of SBO associated with haemostatic agents can be minimised by adhering to proper application techniques and following manufacturer guidance. Baxter, the manufacturer of FloSeal, advises that excess material should be removed after achieving haemostasis to reduce the potential for adhesion formation and obstruction [11, 18]. Ethicon, the producer of Surgicel, highlights that leaving excessive product in situ may lead to foreign body reactions and hinder absorption, particularly when applied near delicate structures such as the bowel [19]. This is particularly important in areas with organs of high mobility, such as the bowel, as they can shift into the space occupied by the retained material, theoretically increasing the risk of adhesion formation and obstruction. In this case, this guidance was not followed: a small amount of FloSeal was deliberately retained because the surgeon judged that irrigating it away risked disturbing the clot and re‐starting bleeding from the raw, off‐plane dissected surface near the duodenum, a pragmatic decision to avoid a potentially worse complication, but one that ran counter to the manufacturer’s instructions. We highlight this as a modifiable technical factor and suggest that any deliberate retention of material be documented in the operative note, with a lower threshold for suspecting SBO if atypical symptoms develop.

This report is subject to the inherent limitations of a single‐case observation. Reporting bias likely also affects the apparent rarity of this complication since uncomplicated instances of retained haemostatic material are not typically reported. These findings should therefore be regarded as hypothesis‐generating rather than as definitive proof of a causal relationship between FloSeal and SBO.

This case highlights SBO as a potential surgical technique‐related complication of haemostatic agent use in colorectal surgery. Reported adverse events directly attributable to FloSeal are uncommon [4–8], and this case likely reflects a preventable lapse in technique, the deliberate retention of material against manufacturer instructions, rather than an intrinsic hazard of the product itself. However, the persistence of gelatin‐based materials can contribute to adhesion formation and obstruction, highlighting the importance of irrigation and appropriate application techniques. Clinicians should maintain a high index of suspicion for SBO in patients with rising CRP levels post‐operatively, ensuring timely diagnosis and intervention.

## Funding

No funding was received for this manuscript. Open access publishing facilitated by the University of Adelaide, as part of the Wiley—Adelaide University agreement via the Council of Australasian University Librarians.

## Consent

Informed consent has been obtained from the patient for publication of this report, including the clinical images, and this consent has been documented. This process including providing the patient with a participant explanatory statement and using a clinical case study report consent form used to document consent, as per our Institution’s policy.

## Conflicts of Interest

The authors declare no conflicts of interest.

## Supporting Information

Additional supporting information can be found online in the Supporting Information section.

## Supporting information


**Supporting Information** Table summarising published cases of small bowel obstruction attributed to retained topical haemostatic agents (FloSeal and Surgicel), including the present case, detailing the agent, index procedure, post‐operative day of onset, imaging findings, treatment and outcome.

## Data Availability

The data that support the findings of this study are available upon request from the corresponding author. The data are not publicly available due to privacy or ethical restrictions.
