# Supplementary material for: FloSeal Related Small Bowel Obstruction: A Word of Caution
Source: Case Rep Surg. 2026 Nov 4;2026:1127650. doi: 10.1155/cris/1127650 (PMC13732559; doi:10.1155/cris/1127650)
Supplement: Supplementary file 1 [file CRIS-2026-1127650-s001.docx]

Table 1. Published cases of small bowel obstruction attributed to a retained topical haemostatic agent.

| **Study** | **Agent** | **Index Procedure** | **Onset (POD)** | **Imaging Findings** | **Treatment** | **Outcome** |
| --- | --- | --- | --- | --- | --- | --- |
| Hobday et al., 2009 | FloSeal® | Laparoscopic gynaecological surgery | 6 | SBO | Re-operation with bowel resection | Recovered after resection |
| Suzuki et al., 2010 | FloSeal® | Laparoscopic gynaecological surgery (n=2) | 3 | SBO, no leak | Re-exploration, adhesiolysis | No resection required |
| Clapp & Santillan., 2011 | FloSeal® | Urology/gynae-oncology/ general surgery (n=3) | 7-9 | SBO, inflammatory reaction at FloSeal® site | Laparoscopic re-exploration | Recovered, no resection |
| Ashraf et al., 2022 | FloSeal® | Laparoscopic appendicectomy | 4 | SBO | Diagnostic laparoscopy, adhesiolysis | Recovered, no resection |
| Bakhit et al. 2019 | Surgicel® | Colorectal cancer surgery | 64 (presented as query tumour recurrence) | SBO, tumour recurrence mimic | Re-operation | Recovered, no resection |
| **Present case** | **FloSeal®** | **Laparoscopic right hemicolectomy** | **3** | **Ileus** | **Laparotomy; adhesiolysis** | **Recovered, no resection** |
